# Complete Genome Sequence of Colistin-Resistant, *mcr-10*-Harboring, Enterobacter cloacae Isolate AVS0889, Recovered from River Water in Switzerland

**DOI:** 10.1128/mra.00165-22

**Published:** 2022-04-27

**Authors:** Michael Biggel, Katrin Zurfluh, Sarah Hoehn, Kira Schmitt, Andrea Frei, Christoph Jans, Roger Stephan

**Affiliations:** a Institute for Food Safety and Hygiene, Vetsuisse Faculty, University of Zürich, Zürich, Switzerland; b Amt für Verbraucherschutz, Steinhausen, Zug, Switzerland; Indiana University, Bloomington

## Abstract

Here, we report the complete genome sequence of colistin-resistant Enterobacter cloacae sequence type 1 (ST1) isolate AVS0889, which was recovered from a river in Switzerland in 2021. The genome consists of a 4.95-Mbp chromosome and five plasmids, including a large plasmid (90.8 kb) harboring a disrupted *mcr-10* gene.

## ANNOUNCEMENT

Following the initial recognition of *Enterobacteriaceae* strains harboring the plasmid-mediated colistin resistance gene *mcr-1* in 2015 (1), nine additional *mcr* (mobilized colistin resistance) variants (*mcr-2* to *mcr-10*) have been observed in various species (2–4). Here, we describe the occurrence of a disrupted *mcr-10* gene in an Enterobacter cloacae isolate from an environmental sample.

E. cloacae isolate AVS0889, showing phenotypic resistance to colistin (MIC of >64 mg/L) and a positive PCR result for *mcr-10*, was isolated in November 2021 from a water sample collected from the river Lorze in Switzerland (coordinates: 47.21537, 8.42497). The water sample (100 mL) was filtered through a 0.45-μm membrane filter (Millipore). The filter was incubated in 10 mL enterobacteria enrichment (EE) broth (BD) at 37°C for 24 h. One loopful of the EE broth was spread on cystine-lactose-electrolyte-deficient (CLED) agar (Oxoid) supplemented with 4 mg/L colistin, 10 mg/L vancomycin, and 5 mg/L amphotericin and was incubated at 37°C for 24 h. Matrix-assisted laser desorption ionization–time of flight mass spectrometry (MALDI-TOF MS) (Bruker Daltronics) was used for preliminary genus identification. Different PCR primers were used to detect *mcr-1* to *mcr-10* genes (5–7). Colistin susceptibility testing was performed by broth dilution, and results were interpreted according to EUCAST breakpoints v12.0 (https://www.eucast.org/clinical_breakpoints). DNA was isolated from a subculture obtained from a single colony that had been grown for 24 h at 37°C on sheep blood agar. Genomic DNA was extracted using the DNeasy blood and tissue kit (Qiagen). Libraries were prepared using the Nextera DNA Flex library preparation kit (Illumina) and sequenced on the Illumina MiniSeq platform (2 × 150 bp). Illumina reads were trimmed with fastp v0.20.1 (8), and quality was assessed using FastQC v0.11.9 (https://www.bioinformatics.babraham.ac.uk/projects/fastqc). For long-read sequencing, libraries were prepared using the SQK-LSK109 kit and sequenced on the MinION platform with a FLO-MIN106 flow cell (Oxford Nanopore Technologies [ONT]). Base calling, demultiplexing, and barcode trimming were performed with guppy v4.2.2 (ONT), and quality was assessed with LongQC v1.2.0 (9). A hybrid assembly was generated from 800 Mbp of long-read data (37,711 reads [read *N*_50_, 11.5 kb]; coverage, 150×) and 320 Mbp of short-read data (2,155,666 paired reads; coverage, 60×) using the Unicycler v0.4.8 pipeline (10), which includes assembly polishing, circularization, and rotation. Species identification and multilocus sequence typing (MLST) were performed using ribosomal MLST (rMLST) (11) and mlst v2.19.0 (https://github.com/tseemann/mlst). Resistance genes and plasmid replicons were identified using abricate v1.0.1 (https://github.com/tseemann/abricate) (coverage, >90%; identity, >90%) with the ResFinder (12) and PlasmidFinder (13) databases, respectively. The genome was annotated using PGAP v2021-01-11.build5132 (14). Default parameters were used for all software unless otherwise specified.

The complete genome of AVS0889 consists of a circular 4,950,718-bp chromosome (rotated to *dnaA*) and the circular plasmids pAVS0889-a (110,606 bp), pAVS0889-b (90,827 bp; IncFIB[K]), pAVS0889-c (4,783 bp), pAVS0889-d (4,307 bp), and pAVS0889-e (2,454 bp; Col [pHAD28]), with a GC content of 54.7%. AVS0889 was identified by rMLST and MLST as Enterobacter cloacae sequence type 1 (ST1). Two chromosomally encoded antimicrobial resistance genes were detected, namely, *bla*_CMH_ (AmpC β-lactamase) and *fosA* (fosfomycin thiol transferase). In addition, plasmid pAVS0889-b harbored a disrupted *mcr-10* gene. This gene contained a 29-bp insertion at position 64 and an IS*26* insertion element inserted near the 5′-terminal end. No complete *mcr* genes were detected, suggesting that the phenotypic colistin resistance of AVS0889 is mediated by one of the various known intrinsic resistance mechanisms (2).

### Data availability.

The complete genome sequence of AVS0889 has been deposited in GenBank under the accession numbers CP092042 (chromosome), CP092043 (pAVS0889-a), CP092044 (pAVS0889-b), CP092045 (pAVS0889-c), CP092046 (pAVS0889-d), and CP092047 (pAVS0889-e) under BioProject number PRJNA803974. The sequencing data were deposited in the NCBI Sequence Read Archive (SRA) under the accession numbers SRR17902521 (Illumina reads) and SRR17902520 (ONT reads).
